# An explainable ensemble for diabetic retinopathy grading with a novel confidence quality factor and configurable heatmaps

**DOI:** 10.1007/s11517-026-03514-2

**Published:** 2026-02-05

**Authors:** Javier Civit-Masot, Francisco Luna-Perejon, Luis Muñoz-Saavedra, José María Rodríguez Corral, Manuel Domínguez-Morales, Anton Civit

**Affiliations:** 1 Robotics and Computer Technology Lab, ETSII, Universidad de Sevilla, Reina Mercedes s/n, Seville, 41018 Spain; 2 Computer Engineering Research Institute, Universidad de Sevilla, Reina Mercedes s/n, Seville, 41018 Spain; 3 Applied Robotics Research Group, School of Engineering, Universidad de Cádiz, Avda. Universidad de Cádiz, 10, Puerto Real, Cádiz, 11519 Spain

**Keywords:** Diabetic retinopathy, Explainable AI (xAI), Deep learning, Explainable ensemble, Medical imaging, ICDR, Clinical decision support

## Abstract

While current artificial intelligence (AI) tools aid in detecting diabetic retinopathy (DR), they face significant challenges that limit their clinical utility. Most are restricted to binary (referable vs. non-referable) screening and operate as “black boxes,” lacking the detailed, transparent explanations required for diagnostic confidence. This study addresses these gaps by introducing a novel, explainable ensemble-based approach for detailed DR grading. Our system utilizes a parallel ensemble of two efficient deep learning networks, EfficientNetV2 and ConvNeXt, to perform a full five-class international clinical diabetic retinopathy (ICDR) classification. The proposed model achieves state-of-the-art performance, with 96.7% accuracy and an Area Under the Curve (AUC) over 96% for all classes on a public dataset. More importantly, it provides a comprehensive diagnostic report designed to enhance clinical trust and utility. This report features multiple, configurable superimposed heatmaps, two probability-ordered diagnostic suggestions, and a novel quality factor that estimates the confidence of the prediction. By offering richer, more transparent, and interactive explanations, our system moves beyond simple screening to function as a valuable diagnostic assistance tool for ophthalmologists and other healthcare professionals.

**Graphical abstract:**

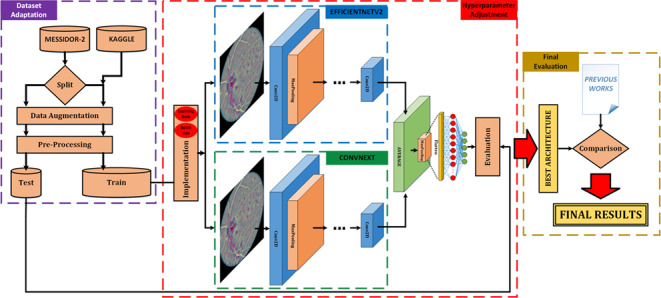

## Introduction

According to Ogurtsova et al. [1], in 2021 537 million adults, that is, 1 in every 10, live with diabetes. This number is expected to increase to 783 million by 2045. More than 75% of adults with diabetes live in low- and middle-income countries. Diabetes was responsible for 6.7 million deaths in 2021 and was responsible for more than 966 billion dollars in health expenditure.

**Fig. 1:**
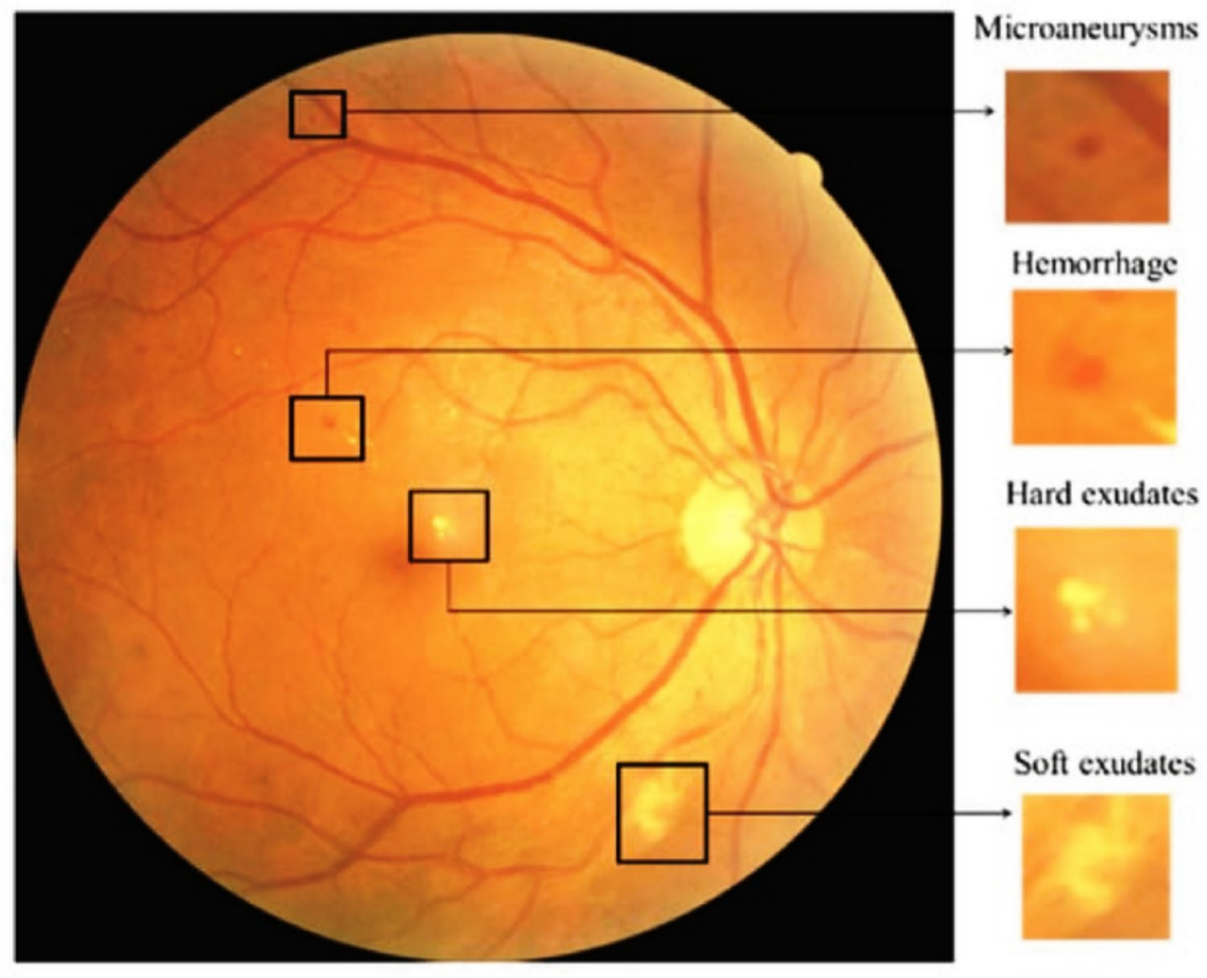
Key pathological lesions that are characteristic signs of diabetic retinopathy (DR). The main fundus image on the left shows several signs of the disease, which are magnified in the insets on the right: microaneurysms, hemorrhages, hard exudates, and soft exudates. The presence and severity of these elements are used to grade DR severity levels [3] CC BY 4.0

Diabetic retinopathy (DR) is a serious eye condition that causes significant vision loss and even blindness in individuals with diabetes. It is caused by damage to the blood vessels in the retina, where the light-sensitive cells of the eye detect light variations and send signals to the brain through the optical nerve. High blood sugar levels associated with diabetes can block small vessels in the retina, causing blood leaks. These may also lead to the growth of new blood vessels that may not function properly and can easily bleed [2]. In Fig. 1, we can see the main lesion caused by DR. Microaneurysms are small capillary bulges and usually are the initial clinical signs of retinopathy. Hard exudates, consisting of lipoproteins that leak from damaged retinal vessels, appear as glossy, yellowish deposits that can form circular patterns around microaneurysms. Haemorrhages are caused by capillary breaks and appear as small superficial dots or larger blots within the deeper layers of the retina. In later stages of DR, due to blocked vessels, soft exudates emerge as pale lesions with indistinct edges.

According to the International Clinical Diabetic Retinopathy (ICDR) [4] severity scale, different levels of DR severity are defined. Level 0 is associated with healthy individuals. Level 1 corresponds to mild nonproliferative DR (NPDR) in which one or a few microaneurysms are present in the retina. Level 2 is associated with moderate NPDR, in which there are numerous microaneurysms and retinal haemorrhages with or without cotton wool spots. Level 3, severe NPDR, is the case in which numerous haemorrhages and microaneurysms are present in all quadrants of the retina, cotton wool spots in 2 or more quadrants, and intraretinal microvascular abnormalities in at least 1 quadrant. Finally, level 4, proliferative DR is an advanced stage of DR, in which new thin and fragile blood vessels are generated, which are at high risk of leakage and can cause severe vision loss and even blindness.

It is important to introduce the concept of referable DR as it is used in many research works. A DR case is considered referable (must be further studied by a retina specialist) when it is level 2 or higher.

DR can be diagnosed through eye examinations. The most common method of diagnosing DR is a dilated eye exam, but several other tests are used to diagnose DR, including [5] fluorescein angiography (FA) where a dye is used to highlight blood vessels in the retina, optical coherence tomography (OCT) that provides high quality cross-sectional images of the retina, and fundus imaging that involves taking photographs of the back of the eye (fundus).

Most AI-based tools are based on fundus photography data. There are several reasons for this, as FA has important side effects and with current image preprocessing techniques, can usually be replaced by FI. Retinal OCT is the best available diagnostic technique, but the equipment required is expensive and rarely available in developing countries.

The design of diagnostic assistance systems for DR capable of extracting characteristics from images and differentiating between those that suggest some type of disease and those that represent a healthy patient [6] provides several benefits: Allows scale screening where cases that are easy to diagnose can be handled by a quick analysis of the AI based report, reduces specialists’ workload leaving more time for difficult cases, and consequently reduces the time needed for diagnosis.

For these reasons, many AI-based medical diagnostic aids have been designed, developed, and evaluated in recent years. These systems provide fast classification, but require large datasets so that, in their training phase, the most relevant features can be correctly extracted from the data. These techniques have obtained very positive results with a correct diagnosis rate greater than 80% [7–9]; even reaching, in several cases, accuracy values greater than 95% [10–12].

It is not uncommon for diagnostic aid systems trained with data from specific sources to produce inferior results when samples from other medical centres are evaluated or when different scanning devices are used [13]. This fact has led to some mistrust in automated diagnostic support systems among healthcare professionals.

Another important problem with DL systems is that the weights of the trained neural network do not provide any information that is understandable by the user and helps to explain the objective criteria used to perform the classification. For this reason, these systems are often known as “black boxes” [14]. To avoid this problem, the use of explainable artificial intelligence (xAI) and explainable deep learning (xDL) technologies has gained significant importance. These technologies provide information on the classification criteria used in the automatic system [15, 16]. The information obtained after these analyses is of great importance, not only for detecting possible classification errors, but also to allow healthcare professionals to understand the decisions made in the correct classifications [17, 18].

Our diagnostic aid system is built upon a parallel ensemble of two distinct deep learning networks: EfficientNetV2 [19] and ConvNeXt [20]. These networks were chosen for their high performance and availability of pre-trained implementations. Unlike some previous approaches, our model does not rely on custom architectures. Instead, we leverage the established capabilities of these networks and enhance them by combining them in a novel ensemble. The ensemble approach is a key innovation, as it allows the system to produce more robust and informative results than a single network approach. The system outputs not only a diagnosis but also a quality factor that is an estimate of the probability ratio between the first and second suggested diagnostic alternatives. This helps clinicians in understanding the confidence of the system’s prediction.

In this work, a diagnostic aid system for DR using explainable deep learning (xDL) techniques is designed, implemented, and tested using fundus imaging. In this way, not only the possible diagnosis results, but also the areas of images the classifier has used to obtain the diagnosis can be provided to the ophthalmologist (together with a detailed classification report on the confidence of the result belonging to each class). As a novelty, the proposed system is based on a parallel ensemble of two networks. We have demonstrated that this architecture significantly improves the explainability of the results and the quality of the report provided to the ophthalmologist over the result obtained by single-network systems. A comparison will also be made with previous work in the area. The main contributions of this work that overcome the limitations of current approaches are:

- **Ensemble Architecture**: We employ a parallel ensemble of two high-performance deep learning networks: EfficientNetV2 [19] and ConvNeXt [20]. This ensemble approach leverages the strengths of both networks to enhance diagnostic accuracy and improve the quality of explanations, which is a significant improvement over single-network systems [10, 21].
- **Explainable Deep Learning (xDL)**: Our system integrates xDL techniques, specifically the Grad-CAM algorithm [22], to generate heatmaps that highlight the areas of the fundus images most influential in the model’s decision-making process. This provides clinicians with visual explanations and increased transparency and trust in the model’s predictions [23, 24].
- **Quality Factor**: We introduce a quality factor that estimates the probability ratio between the first and second diagnostic alternatives. This factor provides clinicians with a measure of the system’s confidence, highlighting cases where the second prediction option should also be considered.
- **Enhanced Performance for standardized five class classification**: Our approach achieves state-of-the-art performance, with a 96.7% accuracy and an AUC over 96% for the five-class International Clinical Diabetic Retinopathy (ICDR) classification problem. This represents a notable improvement over other methods trained and evaluated on the same dataset [25].
- **Customizable Reporting**: The system provides ophthalmologists with configurable heatmaps and two probability-ordered diagnostic suggestions and allows for the selection of heatmaps from individual networks, thus catering to individual preferences in image interpretation. The ensemble architecture provides ophthalmologists with multiple interpretable explanations, recognizing that clinicians may not always prefer the same explanation.
- **Data Augmentation Strategy**: To address dataset imbalances, we implement a combination of techniques including the addition of images from the Kaggle EyePACS dataset [26], ensuring a more balanced training dataset.

The remainder of the manuscript is organized as follows. In Section 2 we discuss previous work related to DR classification and consider the different available DR datasets, as the work can only be fairly compared with other systems that use the same datasets. In Section 3, we discuss the balancing and preprocessing techniques of the dataset, the architecture of the classifier ensemble, the metrics used to evaluate it, the xDL techniques used to produce reports to help the ophthalmologist make the diagnosis. The results obtained after testing the classifier and the Explainable Deep Learning reports are explained in Section 4, together with a comparison with previous work. Finally, Sections 5 and 6 draw the discussion and conclusions of this work and propose future research lines.

## Related works

To be able to establish the state-of-the-art in relation to AI based DR grading systems, we performed initially a search for systematic literature reviews in the field. In the work performed by Islam et al. [27], 20 studies met the inclusion criteria. Analysing these studies, it was found that, for referable DR (i.e., two-class classification), the average area under the receiving operating curve (AUC) was 0.97, the average sensitivity was 0.83, and the specificity was 0.92. These performance parameters are discussed in subsection 3.3. In this review, only one work [28] uses explainability techniques.

In [29] data from 33 studies were analysed reporting sensitivities and specificities of AI tools that ranged from 83·3%–100% to 68·8%–98% respectively for referable DR. This scoping review does not include any explainability-aware solution.

In [30] 15 systems based on DL and 4 systems based on traditional machine learning (ML) are evaluated for referable DR classification. All studies reported sensitivities above 85% while the specificity estimates varied widely, ranging from 20% to 100%. All studies evaluating ML systems reported specificities below 80%, while most DL systems reported specificities well above this value. In this review, no explainable solutions are presented.

It is important to mention that the three mentioned literature reviews include data from studies using different DR datasets. The quality, number of images, and ICDR class distribution of images in these datasets vary very significantly, and, thus, it is very difficult to make any fair comparison between works that use different DR datasets. For this reason, we will discuss the options for the data set in Subsection 2.1 and compare our system with works that use the same dataset in Section 4.

### DR datasets

**Fig. 2:**
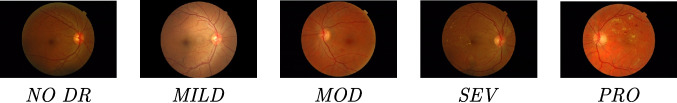
Example fundus images from the Messidor-2 dataset, illustrating the five severity levels of the ICDR scale used for classification in this study. From left to right: No DR (Level 0), Mild (Level 1), Moderate (Level 2), Severe (Level 3), and Proliferative (Level 4)

According to Gupta et al. [31], there are two possible approaches to building a DR dataset. The first approach is to provide a set of images that includes blood vessel segmentation data. This approach is used by Drive, Stare and Chase [32]. The alternative approach is based on manual labelling of the lesions and the provision of an expert-verified severity level of DR. This approach is followed by Messidor, Messidor-2 and Kaggle DR detection dataset [27]. Messidor [25] consists of 1200 images obtained with similar instrumentation from three different departments of ophthalmology. The images are classified using a variation of the ICDR classification that uses very similar levels from 0 to 4 and includes independent parameters on macular oedema and neovascularization. Messidor-2 [33] includes 1,788 macula-centered fundus images obtained without pharmacological dilation using a Topcon TRC NW6 non-mydriatic camera with a 45 degree field of view (see example images in Fig. 2. This provides very good quality images that are typical of what is currently available in most ophthalmological clinics. For this reason, Messidor-2 was selected for our work. The DR grade distribution of the images in the Messidor-2 dataset is shown in Table 1.

**Table 1:** Distribution of the five diabetic retinopathy severity grades in the original Messidor-2 dataset. The table highlights the significant class imbalance present in the data, which motivates the balancing and augmentation techniques used in this study

Grade	Images	Fraction
[0] No DR	1017	58.30%
[1] Mild	270	15.40%
[2] Moderate	347	19.80%
[3] Severe	75	4.30%
[4] Proliferative	35	2%

### Related works

Previous work that could be directly compared with our study was found using a dual approach. First, the search for systematic reviews of the publications on AI-based DR classification, already mentioned in Section 2, was carried out. In this search, we found four useful results. Shahriari et al. [34] provided no useful reference, as it does not include any work that uses the Messidor-2 dataset. In the works [27, 29, 30] we found the references included in Table 2. To further complement this approach, a search was performed in the main search engines for research papers (Scopus, IEEExplorer, and Google Scholar (GS) with the keywords: "Mesidor-2", “Diabetic Retinopathy”. After analysing the papers and excluding preprints or arXiv/bioRxiv works, we selected the works included in the GS row of Table 2.

**Table 2:** Summary of the literature search for identifying previous works that use the Messidor-2 dataset for evaluation. The table lists the primary studies found and categorizes them by their origin, either from existing systematic reviews or a direct literature search

Source	Works
Islam et al. [27]	Pires et al. [35],
	Gargeya et al. [36],
	Abràmoff et al. [33]
Cleland et al. [29]	Gulshan et al. [37]
Zhelev et al. [30]	Abràmoff et al. [38]
Literature Search	Lahmar et al. [39],
	Voets et al. [40],
	Yaqoob et al. [41],
	Zhang et al. [42],
	Papadopoulos et al. [43]

The ten selected works are presented below with a brief summary. In those cases where high-sensitivity and high-specificity operating points are provided, we use the data for the high-sensitivity case.

Pires et al. [35] use a custom architecture based on o_O [44] and Vgg16. The system, which performs two-class classification between referable and non-referable cases, was trained with the Kaggle EyePACS dataset and tested on Messidor-2 obtaining an AUC value of 98.2%. The work does not provide data on any other evaluation metric.

In the work performed by Gargeya et al. [36] a custom network is used to perform a two-class classification between noDR and all DR classes. The system was trained on 75137 publicly available images and obtained a 94% AUC value on the Messidor-2 dataset.

In the work performed by Abràmoff et al. [33], previously available traditional image processing techniques were used to detect specific lesions related to DR and then combined to detect referable DR. The system achieved an AUC of 93.5% with a sensitivity of 96.5% and a specificity of 59.4%. The authors later improved their work using a set of CNNs to detect lesions and obtained a 98% AUC with a sensitivity of 96.8% and a specificity of 87% [38].

Zhang et al. [42] trained a Graph Convolutional Neural Network with more than 12000 images, of which only 500 were manually labelled. The system achieved a 97. 4% AUC with 91. 8% accuracy, 90. 2% sensitivity, and 93% specificity in the Messidor-2 dataset. Although it is not fully clear in the paper, the results seem to be associated to a 5 class ICDR classifier.

A dominant approach in the literature involves training a single, deep end-to-end network on a large dataset of fundus images. The seminal work by Gulshan et al. [37] demonstrated the power of this method, using the Inception-v3 architecture on a private dataset of over 128,000 images to achieve a near-perfect AUC of 0.99 on the Messidor-2 validation set. This high performance was attributed not only to the deep learning model but also to the extensive, high-quality reference standard established by a large panel of ophthalmologists who graded each image multiple times. The critical importance of the training data and reference standard quality was later highlighted in a reproduction attempt by Voets et al. [40]. Using the same architecture but training on the publicly available (and singly-graded) Kaggle dataset, they were unable to replicate the original results, achieving a significantly lower AUC of 0.853 on Messidor-2. This underscores the challenge of generalizing from private, heavily curated datasets and frames the context for our work, which also uses public data.

In contrast to purely end-to-end models, other researchers have explored alternative architectures to improve performance and interpretability. Abramoff et al. [38] developed a *hybrid* system where multiple CNNs are used specifically as high-performing *lesion detectors*. The outputs of these detectors are then integrated into a classical system to make a final referral decision, an approach designed to avoid the spurious correlations that can arise from training on whole images. On the Messidor-2 dataset, this deep learning-enhanced hybrid system achieved an AUC of 0.980, significantly improving the specificity over their prior non-deep-learning version [33]. Taking a different path, Papadopoulos et al. [43] proposed a *patch-based* multiple-instance learning (MIL) framework. Their model uses an attention mechanism to identify and focus on the most informative patches within an image, thereby achieving both high performance (AUC of 97.6%) and built-in interpretability by generating lesion-focused heatmaps directly from the attention weights. These architecturally distinct approaches provide important context for our work, where we leverage an ensemble of two different *end-to-end* networks to enhance performance and provide multiple, configurable post-hoc explanations, rather than relying on a lesion-first or patch-based design. Other works, such as the comprehensive study by Lahmar et al. [39], have benchmarked several standard architectures, finding DenseNet201 to be the most accurate on Messidor-2 for referable DR classification, further informing the landscape of available models.

It is important to mention that all the analysed works provide data for a dual class DR classification system while we implement and study a full five class ICDR classification system for this reason we will also train our system as a dual class classifier and provide the corresponding results.

## Methods

This section presents tools and systems designed to detect DR and evaluate the results. To do so, we will first present the DR severity scale, then detail the dataset used, its structure, and the pre-processing applied to it. Then, the characteristics of the developed classifiers as well as the justification for their choice will be presented. Finally, we will explain how these classifiers have been evaluated and what information is provided to the pathologist.

**Fig. 3:**
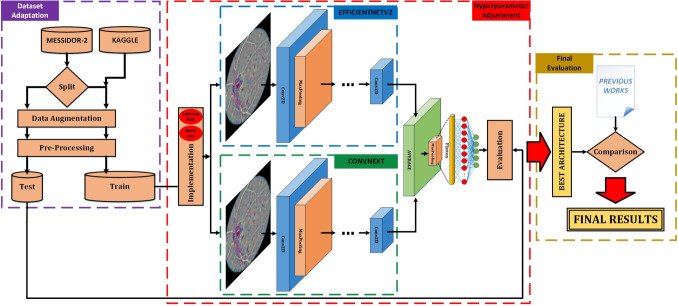
Graphical summary of the experimental workflow. The process begins with dataset adaptation, involving data augmentation and pre-processing of the Messidor-2 and Kaggle datasets. The core of the methodology is the implementation of a parallel deep learning ensemble, whose performance is evaluated and compared against previous studies to yield the final results

In summary, the graphical abstract of the work presented can be seen in Fig. 3.

**Fig. 4:**
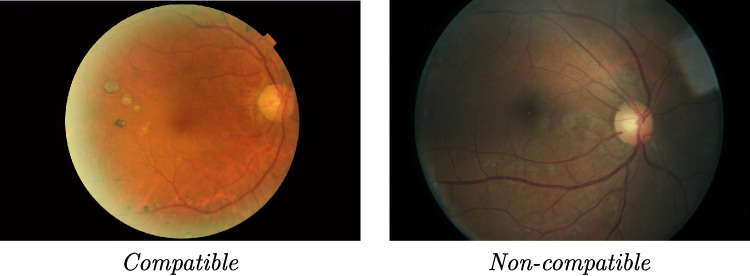
Illustration of the image selection process from the Kaggle EyePACS dataset. Only ’Compatible’ images (left), which feature a complete circular fundus view similar to the Messidor-2 dataset, were selected to augment the training data. ’Non-compatible’ images (right), with different visual characteristics, were excluded to ensure consistency

### Dataset balancing and pre-processing

The main problem found in the Messidor-2 dataset is class imbalance. Any DL classifier tries to maximize the number of samples it classifies correctly during its training process. Therefore, in the case of this dataset, it will favour correct results for the NO DR class. As we have to correctly classify all classes, it is necessary to apply preprocessing techniques to the dataset to improve the distribution of images of each class.

As indicated above, the dataset has a very significant imbalance problem. The most populated class has more than 30 times the number of images of the least populated class. To solve this problem, we use two different techniques. First, data-augmentation techniques are applied offline on the dataset to increase the number of images of the least populated classes. However, the least populated class has only 35 images, and applying data augmentation techniques on it to reach the number of images of the most populated class (NO DR with 1017 images) would imply obtaining more than 28 artificial images for every real image. If this process were carried out, it could condition the results of the classifier, as the augmented images would have characteristics similar to the original ones, and this fact would hinder the classifier performance. To further increase this problem, we have to split the dataset before performing data augmentation, otherwise we would have images derived from the same original image in both the training and the validation datasets. In our case, using an 80%/20% training validation ratio, this means that for the least populated class we have 28 images in the training data set and 7 in the validation dataset.

This is the reason why, in order to balance the dataset, it was decided to use only 150 images for each class. In this way, only slightly more than four augmented images would be needed for each real image of the PRO class and slightly more than one augmented image per each real image for the SEV class. To obtain the new images, a random horizontal flip, a random rotation (between -10 and 10 degrees), and a random RGB channel shift of up to 10 levels (in a 0 to 255 scale) are applied offline to classes needing augmentation, until the number of 150 images for these classes is reached.

As the number of original images in the PRO and SEV classes is very small, using only these techniques did not provide enough original images in the training dataset to obtain good classifier results. To solve this problem, we used a variation of the solution proposed by [45] or [46] by adding images (obtained with acquisition arrangements similar to those of Messidor-2) from the Kaggle EyePACS dataset to our training dataset (see Fig. 4). These images are easy to identify, as they include a complete circular fundus image with an angular position small square mark in the first quadrant. Even though these images are very similar to those in the Messidor-2 dataset they were added to all classes to avoid the possibility of modifying the characteristics of the less populated classes. It is important to mention than in a real clinical scenario analyzed fundus images would come from similar acquisition devices and, thus, the system should be designed to maximize its performance for images acquired in this situation. Table 3 shows, for the different classes, the original number of images in the Messidor-2 dataset, the number of these images that were randomly selected, the distribution of these images between the training and validation datasets and the number of compatible images that were added from Kaggle EyePACS to the training dataset. With the addition of these images, only about a third of the training images in the less populated class come from data augmentation.

After the initial data augmentation process, the images are cropped to circles to reduce border effects, resized to (600, 600, 3) and a variation of the widely used weighted Gaussian blur algorithm is applied [26]. For each image produced by the initial augmentation process, we produce three images with different values of the Gaussian kernel standard deviation. Several other alternatives, including color balancing, adaptive histogram equalization, and combinations of these techniques with the finally used ones, were evaluated with inferior results for the proposed network architecture than for the selected solution. During the training phase, images are generated by an image data generator with characteristics similar to those obtained by static data augmentation. This dynamic augmentation approach further increased the diversity of the training data, contributing to the improved performance of the model. These measures not only improve the model’s ability to generalize to new images of DR, but they also help to address the problem of having fewer samples from the more severe classes of DR, which can be very rare. Table 4 shows the total number of images obtained by static augmentation (second row) and dynamic augmentation (third row) for a 30-epoch training process. The data contained in Table 4 are related to the most populated classes. The number of images would be reduced by 20% for the severe class and by 30% for the proliferative class.

**Table 3:** Composition of the initial dataset for each class prior to data augmentation. The table details the number of images selected from the primary Messidor-2 dataset, the split of these images into original training and validation sets, and the number of compatible images added from the Kaggle EyePACS dataset to supplement the training data for class balancing.

Class	Messidor-2	Selected	Orig. Train	Orig. Val	Kaggle train
NoDR	1,017	150	120	30	40
Mild	270	150	120	30	40
Moderate	347	150	120	30	40
Severe	75	75	60	15	70
Proliferative	35	35	28	7	80

While training and testing the proposed model on a larger, more diverse dataset, such as the Kaggle EyePACS dataset, would be interesting for a comprehensive evaluation and to ensure generalization [26], the current study focuses on maximizing performance when using the same acquisition instrument and has been also constrained by the need for a direct comparison with existing literature that follows this approach which primarily uses the Messidor-2 dataset [25]. Given these limitations, a pragmatic approach was adopted, leveraging a subset of the EyePACS dataset to augment the Messidor-2 dataset, which was used as the primary dataset for this work. The selection of images from the EyePACS dataset was based on their compatibility with the Messidor-2 dataset, specifically targeting images acquired with similar fundus camera arrangements. This ensured that the added images would complement the Messidor-2 images without introducing significant variations due to differing acquisition methods. To minimize potential bias from the addition of these external images, a careful selection process was followed. Only images exhibiting a complete circular fundus view, along with the characteristic small square mark in the first quadrant (as shown in Fig. 4 of the main text), were selected. These specific criteria ensured that the augmented images closely matched the characteristics of the Messidor-2 images in terms of quality and appearance. Furthermore, as detailed in the methods section, data augmentation techniques were applied to these images to match the distribution and variability of the Messidor-2 dataset, mitigating any unintended introduction of bias to the training data. This approach allowed for a more balanced representation of all DR severity classes in the training dataset, while maintaining a level of consistency with prior works by utilizing Messidor-2 as the core dataset. This decision is made also to compare our results with other works that use Messidor-2 to make a more valid comparison. Future work will include training the proposed methodology using the full compatible EyePACS dataset.

**Table 4:** Impact of the data augmentation strategy on the total number of images used during a 30-epoch training process. The table shows the initial number of images without augmentation, the increase after applying static (offline) augmentation, and the total number of images generated through dynamic (on-the-fly) augmentation during training. These values correspond to the most populated classes (No DR, Mild, and Moderate)

Dataset	Training images#	Validation images#
No augmentation	160	30
Static augmentation	480	90
Dynamic Augmentation	14,400	2,700

### Classification system

Our initial approach was to use the same classifiers as those used by Muñoz-Saavedra et al. [47] or by Civit-Masot et al. [48]. It soon became clear that none of these architectures (EfficieNet-B0, Vgg16, ResNet50 or low computational cost custom CNNs) was adequate either by itself or as part of an ensemble was able to obtain state-of-the-art performance for the 5 class DR grading problem. Some of these networks have been used in previous work. In [49], the authors evaluated Vgg16 and Resnet50 using the Kaggle EyePACS dataset and obtained accuracy values of 25% and 70%, respectively. As another example, Rajkumar et al. [50] also uses a pre-trained ResNet50 network and reports an accuracy of 89.4%. Although this result does not seem inadequate, the authors provide the corresponding confusion matrices, and it can be seen that the results are obtained on a very unbalanced dataset and over 90% of the correct predictions correspond to the NO DR class. From the analysis of existing SOA (state-of-the-art) solutions (discussed further in Section 2.2 we can see that successful architectures work with resolutions higher than the typical (224, 224, 3) used in ImageNet pretrained Vgg16, ResNet-50 or EfficientNetV0. To make these higher resolution networks efficient, more advanced architectures are needed.

Although we have tested some custom networks (e.g. variations of those proposed in [48] or of the downward path of the recursive U-Net implementation used in [51], these implementations have not led to SOA results. Thus, we have settled for current generation networks that provide ImageNet-pretrained implementations with image resolutions greater than (352,352,3) as this value or greater is used in several successful implementations [34]. Both EfficientNetV2 [19] and ConvNeXt [20] are modern high-performance networks that provide pretrained implementations with the required resolutions. EfficientNetV2 and ConvNeXt represent two distinct yet significant advancements in convolutional neural network design. EfficientNetV2 focuses on achieving faster training speeds and improved parameter efficiency while maintaining high accuracy, building upon the compound scaling principles of its predecessor. It employs a combination of training-aware neural architecture search (NAS) and scaling, emphasizing smaller model sizes and utilizing operations like fused-MBConv to reduce memory access overhead. In contrast, ConvNeXt aims to modernize a standard ResNet, bridging the gap between convolutional networks and Vision Transformers. Its design is centered around incorporating key architectural elements from Transformers, such as larger kernel sizes, layer normalization, GELU activations, and an inverted bottleneck structure, demonstrating that purely convolutional models can achieve state-of-the-art performance while preserving the inherent inductive biases of ConvNets. ImageNet 21K accuracy, together with the number of parameters and computational requirements, are shown in Table 5 for the implementations of the proposed networks that meet our requirements. As we are looking for computationally efficient implementations, our initial choice has been to select EfficientNetV2M and ConvNeXt-B for our implementation of the DR classification tool. These models provide similar ImageNet accuracy, but it is clear that ConvNeXt-B requires more computational power and has a greater number of parameters. In Section 4 we will present compelling reasons to retain both networks and combine them in an ensemble.

The system uses Grad-CAM [22] to generate heatmaps, highlighting the areas of the image most important to the classification. The ensemble nature of the network means that there is no single final convolutional layer, and so the heatmaps are produced for both EfficientNetV2 and ConvNeXt. The final report can show either the averaged ensemble Grad-CAM or individual Grad-CAMs, which can be chosen based on the preference of the clinician. The option to choose between the different heatmaps is also a novel feature, as ophthalmologists do not always prefer the explanation given by the same network.

**Table 5:** Comparison of candidate network architectures for the proposed ensemble. The table shows the ImageNet Top-1 accuracy, number of parameters, computational cost (FLOPs), and input resolution for models from the EfficientNetV2 and ConvNeXt families. Based on this comparison, EfficientNetV2M and ConvNeXt-B were selected for the final implementation.

Model	Top1	Params	FLOPs	Input
EffV2M	86.2	54.1M	24G	480
EffV2L	86.9	119.5M	53G	480
EffV2XL	87.2	206.8M	94G	512
ConvNeXt-B	86.8	89M	45G	384
ConvNeXt-L	87.5	198M	101G	384

### Classifier evaluation

To evaluate the effectiveness of the classification system we follow the guidelines presented in [52] and use metrics widely used in ophthalmology diagnosis related applications: accuracy (the most widely used metric), sensitivity (also known as recall), specificity, precision, and $\mathrm{F}1_{score}$ [53]. To this end, the classification results obtained for each class are tagged as “True Positive” (TP), “True Negative” (TN), “False Positive” (FP) or “False Negative” (FN). According to them, the high-level metrics are presented in the following equations.

1 $$\begin{aligned} Accuracy = \sum _{c}\frac{TP_c + TN_c}{TP_c + FP_c + TN_c + FN_c}, c \in classes \end{aligned}$$

2 $$\begin{aligned} Specificity = \sum _{c}\frac{TN_c}{TN_c + FP_c}, c \in classes \end{aligned}$$

3 $$\begin{aligned} Precision = \sum _{c}\frac{TP_c}{TP_c + FP_c}, c \in classes \end{aligned}$$

4 $$\begin{aligned} Sensitivity = \sum _{c}\frac{TP_c}{TP_c + FN_c}, c \in classes \end{aligned}$$

5 $$\begin{aligned} F1_{score} = 2*\frac{precision * sensitivity}{precision + sensitivity}. \end{aligned}$$

About those metrics:

- Accuracy: all samples classified correctly compared to all samples (see Eq. 1).
- Specificity: proportion of “true negative values ” in all cases that do not belong to this class (see Eq. 2).
- Precision: proportion of “true positive” values in all cases that have been classified as such (see Eq. 3).
- Sensitivity (or Recall): proportion of “true positive” values in all cases that belong to this class (see Eq. 4).
- $\mathrm{F}1_{score}$: This indicator considers both the precision and the sensitivity (recall) of the test. It is the harmonic mean of both parameters (see Eq. 5).

These metrics are widely used in DL systems; however, in ophthalmological diagnostic systems, other quality indicators, such as AUC, are used. This indicator is the area under the ROC curve (Receiver Operating Characteristic) [52] that is the visual representation of the True Positives Rate (TPR) versus the False Positives Rate (FPR) for different classification thresholds. A possible interpretation of the AUC is as the probability that the model ranks a random positive example more highly than a random negative example.

Therefore, the classifier systems developed in this work will be evaluated according to the metrics detailed in this subsection.

The proposed diagnostic assistance tool was implemented in Tensorflow/Keras and tested in a Google Colab local environment running on a 64GB DRAM Intel 12th generation i9 with an NVIDIA 1080Ti GPU.

### Report generation

Grad-CAM (Gradient-weighted Class Activation Mapping) [22] algorithm is widely used in medical imaging for several reasons:

- Interpretability and Clinical relevance: Grad-CAM generates heat maps that highlight the regions of an image that are most influential in the decision-making process of the model. This information is valuable in clinical practice [54, 55], helping in the interpretation of images. This additional information also helps to understand how the underlying deep learning network makes decisions and thus improves the trustfulness of the model.
- Model Improvement: Using Grad-CAM, researchers and practitioners can identify areas of the image where the model is having problems producing the correct results. This information can be used to refine the model architecture and improve its performance and, as a consequence, its diagnostic capabilities.

Grad-CAM can be adapted to classification problems (as in this work), visual question answering, and captioning. It uses the gradients of any target concept flowing into the CNN final convolutional layer to produce a coarse localization map that highlights the regions in the image that are more significant for predicting the concept.

**Fig. 5:**
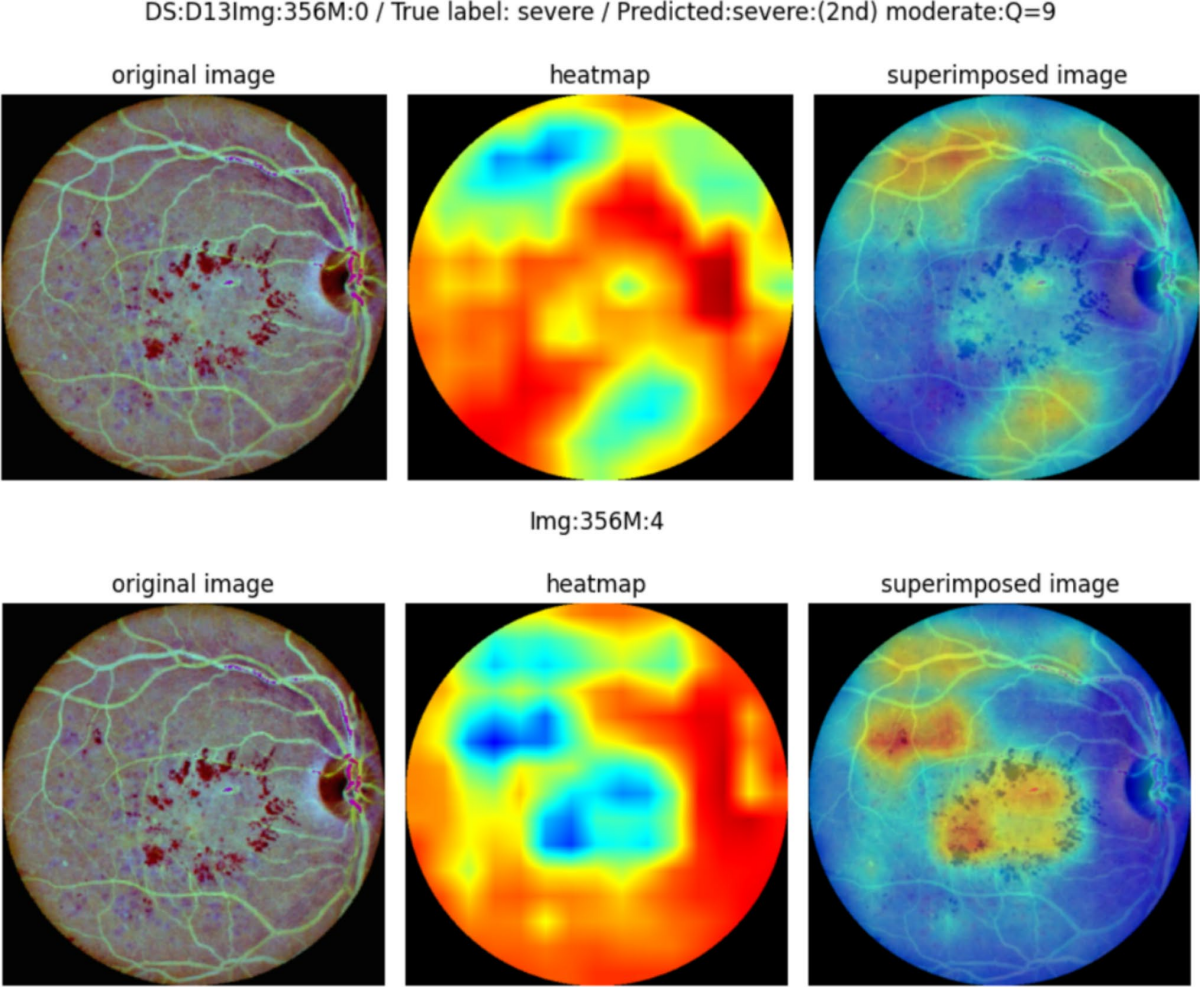
System’s final report given to the healthcare professional. The report includes alternative explanations that can be configured by the practicing ophthalmologist. The interpretation on the top focuses on highlighting healthy areas while the one in the bottom highlights lesions

To provide interpretability for our tool decision suggestions, the information provided to the healthcare professional is completed with an explanation of the proposed decision based on the use of the Grad-CAM algorithm on the evaluated image, and a text report on the confidence of the system regarding the two most probable output classes (obtained from the values produced before the final softmax layer). A summary of the final report generation process is shown in Fig. 5.

Based on this report, the healthcare professional can make the final informed decision, which could be to validate the proposed results or to proceed to a more detailed human-based study of the image and other complementary tests.

Due to the ensemble nature of the proposed network, there is no single final convolution layer, but different final layers for efficientNet V2M and ConvNeXt-B. In ConvNeXt the final result comes from the addition of the results of two final stages. By default, the report shows the averaged ensemble Grad-CAM which is the average of the Grad-CAM heatmaps for both networks considering that, in the case of ConvNeXt the heatmap is also the average of the heatmaps for the convolution layers that are added to produce the results. In a small test with practicing ophthalmologists, who were very satisfied with the usefulness of the provided heatmaps, we have observed that in some cases they preferred the explanation given by one of the individual Grad-CAMs, and thus the option to add additional heatmaps to the report is provided. As a clear example in Fig. 5, ophthalmologists prefer the result in the lower row (M: 4 - produced by efficientNet) to the global averaged result in the upper row (M: 0). One of the main reasons for the attractiveness of the ensemble architecture is the fact that ophthalmologists do not always prefer the explanation from the same network.

In the results section, we will provide further examples of reports corresponding to other classes.

## Results

The results presented in this section demonstrate the performance of our ensemble-based, five-class diabetic retinopathy (DR) grading system. It is critical to contextualize these findings: unlike the majority of previous works that focus on binary classification for referable DR screening [35–38], our model performs a more challenging and clinically informative five-class ICDR grading suitable for detailed diagnosis. The following subsections will not only show the model’s high accuracy on this complex task but also highlight the significance of its novel features, such as the multi-faceted explainability and the quantitative “quality factor,” which together represent a substantial step forward in developing trustworthy clinical decision support tools. We used a five-fold cross-validation analysis and present the results from the worst case. The additional samples from the Kaggle dataset are always included in the training dataset.

**Fig. 6:**
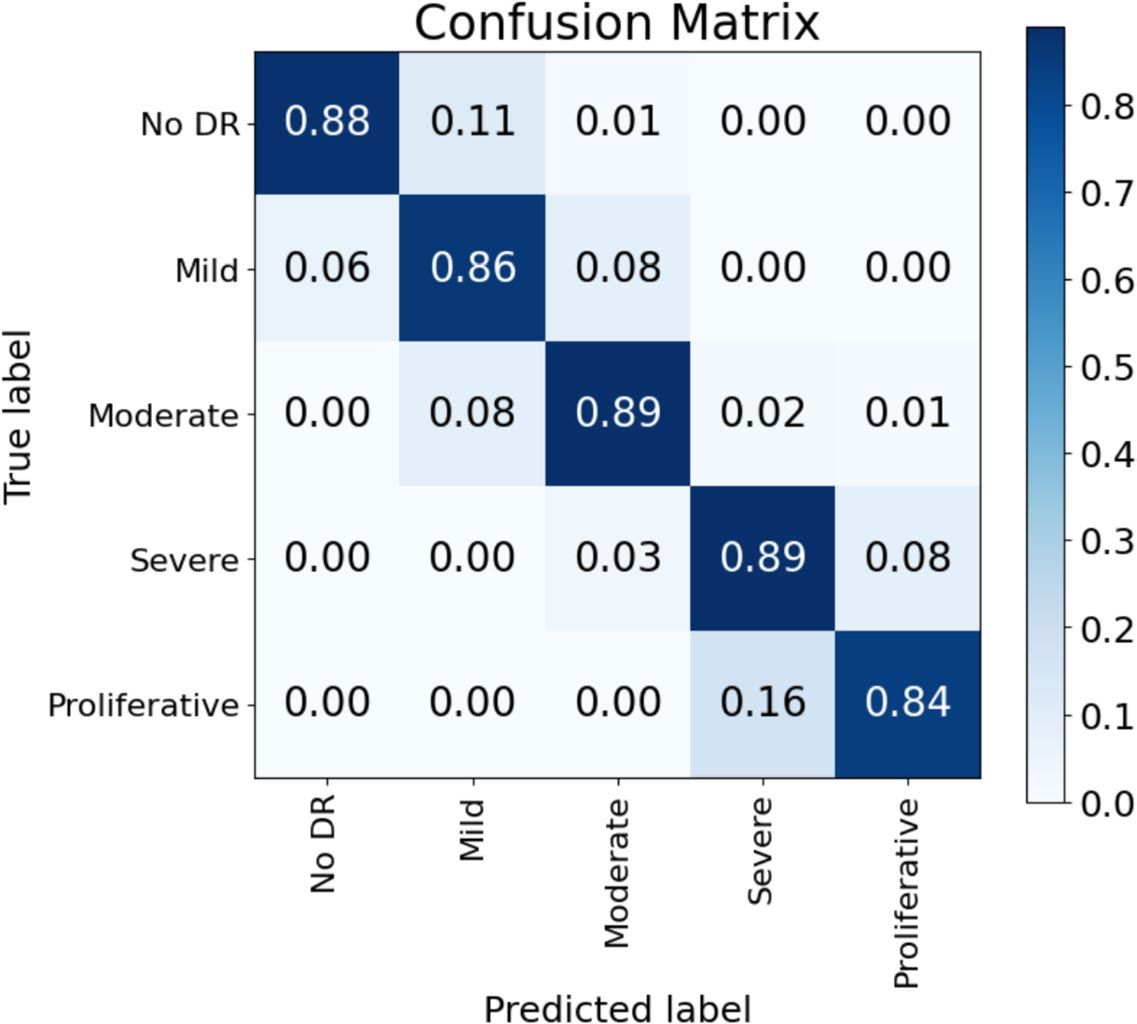
Normalized confusion matrix for the five-class ICDR classification on the validation dataset. The diagonal values demonstrate high accuracy for each class. Notably, the off-diagonal values show that misclassifications occur almost exclusively between adjacent severity levels (e.g., ’Mild’ predicted as ’Moderate’), highlighting the model’s clinically reliable behavior

The confusion matrix for the validation dataset, shown in Fig. 6, reveals a crucial characteristic of our model’s performance. A key finding is that classification errors are almost exclusively confined to adjacent classes (e.g., predicting ‘Severe‘ instead of ‘Proliferative‘ or ‘Moderate‘ instead of ‘Mild‘). This result is highly significant from a clinical standpoint, as it indicates the model’s predictions are robust and diagnostically reliable. Such a predictable error profile minimizes the risk of a patient with severe disease being misdiagnosed as healthy, which is a critical safety feature for any diagnostic aid and a clear advancement in building clinical trust.

The primary focus of this study is to demonstrate the effectiveness of the novel ensemble architecture for diabetic retinopathy grading, emphasizing enhanced explainability and diagnostic accuracy rather than the optimization of individual model components. However, an ablation study was performed by removing each of the ensemble networks. In each case we obtained, as expected, lower performances than those for the combined network. In all the cases the proliferative class was the one that was more frequently wrongly classified. For the more complex ConvNeXt network the class was correctly classified in 81% of the cases, in 17% was classified as Severe and 2% as moderate. For the simpler efficientNet V2M it was correctly classified in 80% of the cases and the remaining 20% were classified as Severe. The behavior for the rest of the classes follows similar patterns. In some classes like No DR and Moderate the performance of the individual networks is very similar to that of the ensemble.

**Fig. 7:**
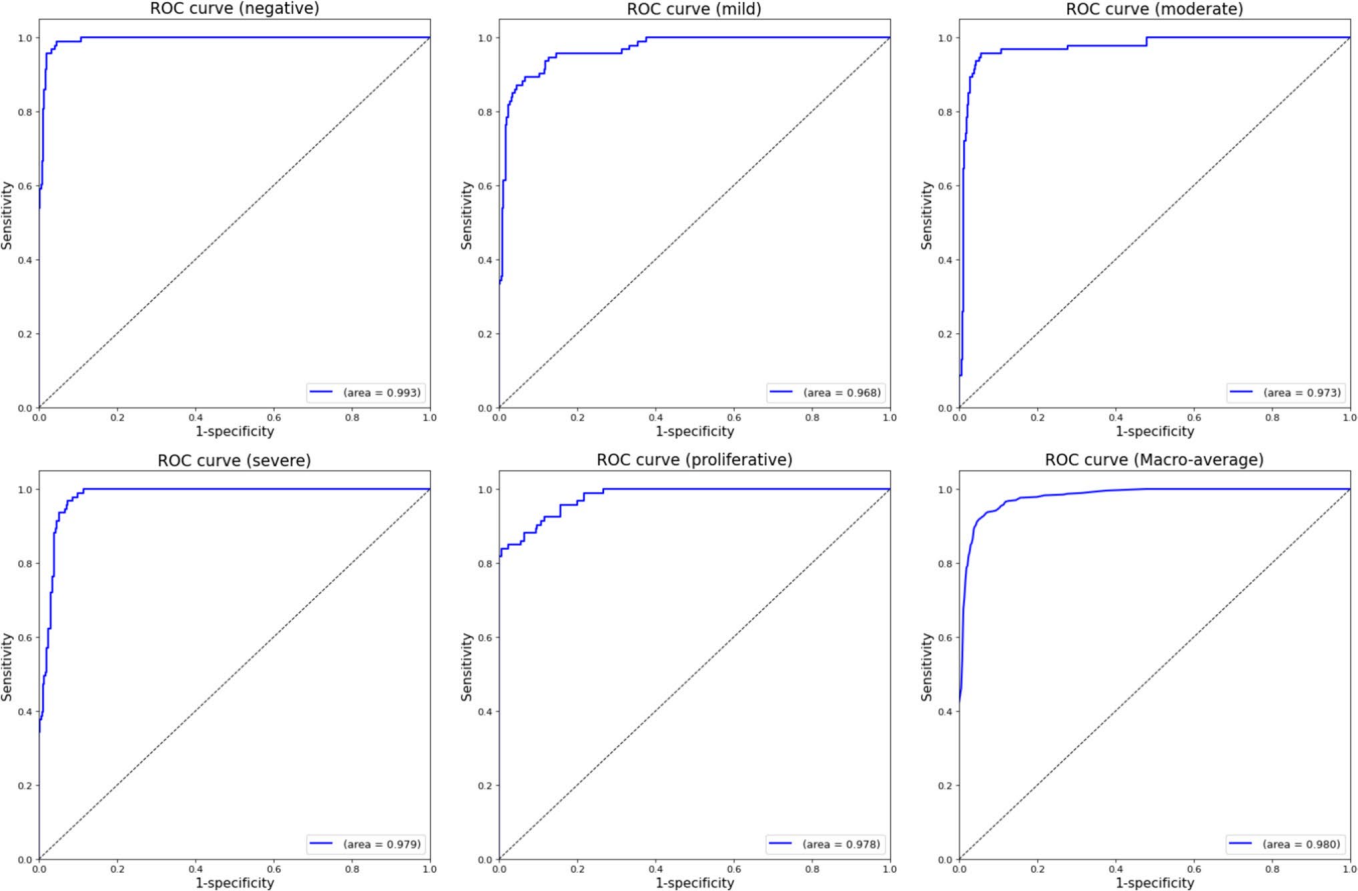
Receiver Operating Characteristic (ROC) curves for the five-class ICDR classifier. The figure displays individual ROC curves and the Area Under the Curve (AUC) for each of the five severity classes, along with the macro-averaged ROC curve representing overall model performance. The high AUC values across all plots demonstrate the model’s robust discriminative ability for each stage of diabetic retinopathy

The ROC characteristics of the different ICDR classes together with their associated AUCs are shown in Fig. 7. It can be seen that the MILD and PRO classes are the most difficult to classify.

### Significance of the explainability report and quality factor

**Fig. 8:**
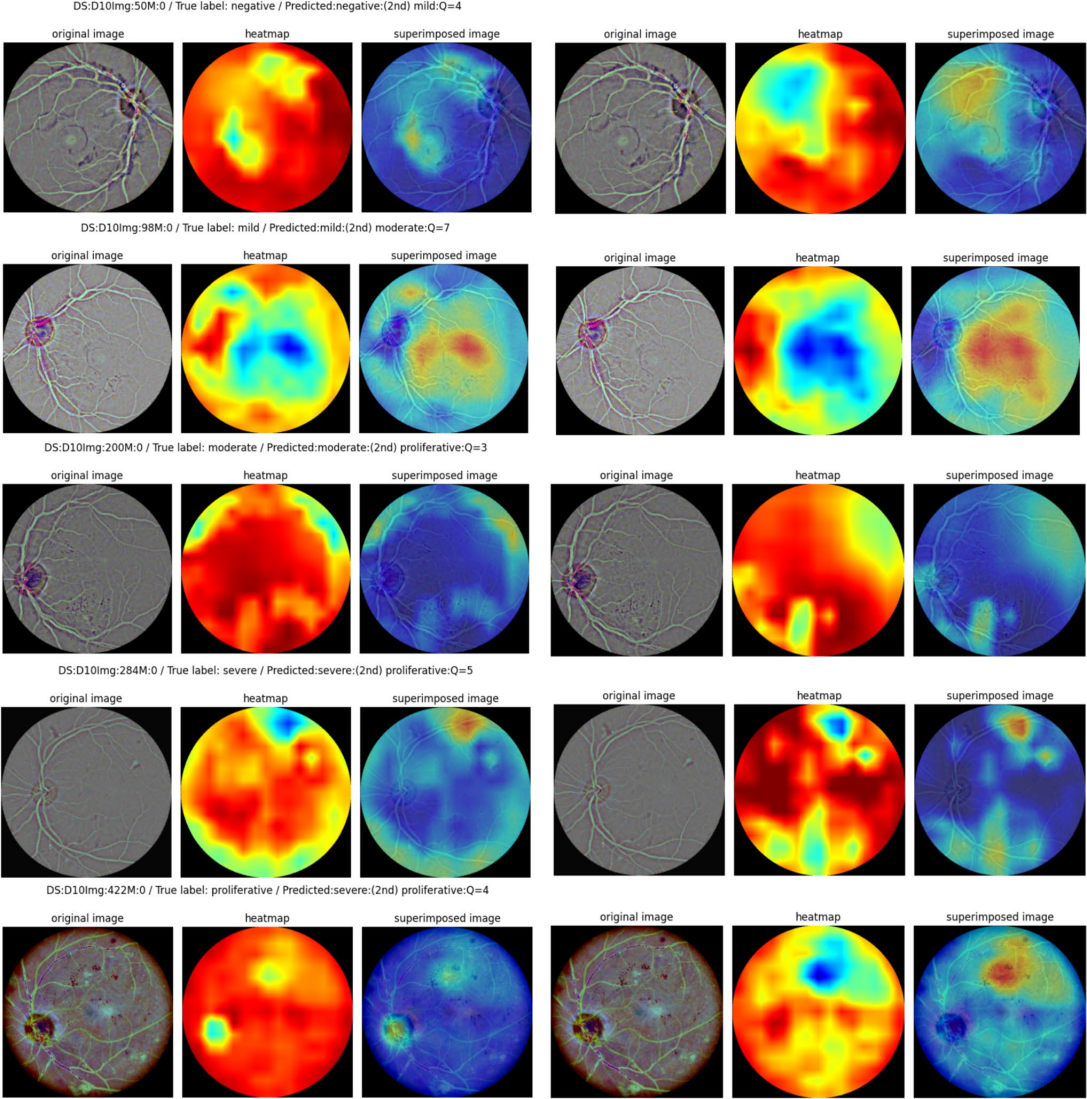
Example report for different ICDR grades. From top to bottom: NoDR(0), Mild(1), Moderate(2), Severe(3) and Proliferative(4). Alternative explanations highlight healthy or problematic areas in a configurable fashion

Beyond classification accuracy, the primary contribution of our work is the generation of a clinically insightful and interactive diagnostic report. A key advancement is the system’s ability to provide **multiple, alternative explanations**, which is crucial because a single explanation is often insufficient for clinical interpretation. This is demonstrated in Fig. 8, which shows example reports for different ICDR grades. The report can display heatmaps from the combined ensemble as well as from individual networks like ConvNeXt. This flexibility is a novel feature that moves beyond the static, single-explanation systems common in the literature.

The value of these multiple explanations is evident in the “moderate” case shown in Fig. 8 (row 3). Here, the system correctly predicts “moderate” with high confidence (Q=4), but its second suggestion is “proliferative”. The ConvNeXt heatmap provides a clear rationale for this, highlighting an area with proliferative characteristics that might otherwise be missed. This ability to present alternative, insightful evidence is a direct result of the ensemble architecture and a significant benefit for clinicians.

Furthermore, we introduce the **“quality factor”** as a quantitative measure of the system’s confidence. This factor, an estimate of the probability ratio between the first and second diagnostic alternatives, provides an immediate, understandable indicator of certainty. A high factor provides greater confidence, while a low quality factor correctly signals to an ophthalmologist that a case is borderline and requires closer inspection.

**Fig. 9:**
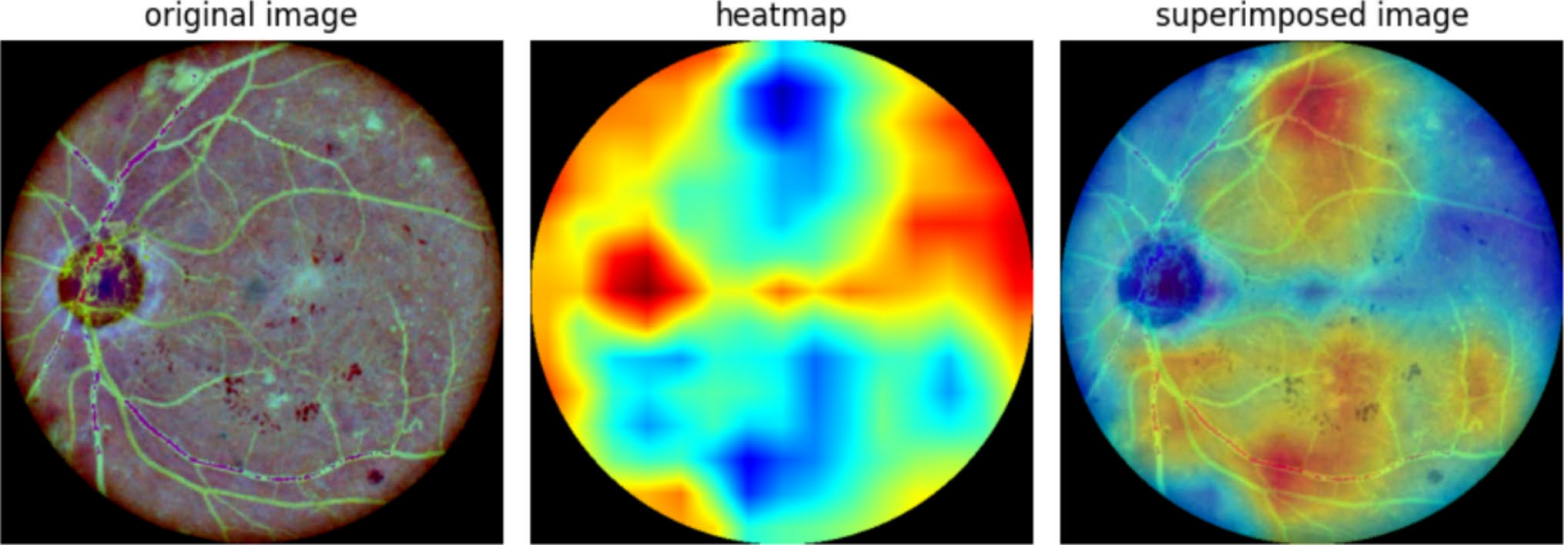
Incorrect prediction. Proliferative predicted as Severe (Q=2). The low quality factor highlights the system’s uncertainty, flagging the case for expert review

This quality factor is also critical for interpreting incorrect predictions. As noted from Fig. 6, the most common classification error is predicting a “proliferative” case as “severe”. Figure 9 shows an example where the system makes this error, but the low quality factor (Q=2) correctly highlights the system’s uncertainty. From a clinical perspective, this type of adjacent-class error is less critical than a major misdiagnosis, and the low confidence score appropriately flags the case for expert review. Similarly, when the system incorrectly classifies a healthy case as “mild DR,” it almost always presents “healthy” as the second option with a low quality factor, again providing a crucial safeguard.

Together, these features transform the system from a simple classifier into a more nuanced and trustworthy decision-support tool.

## Discussion

Next, we analyze the performance metrics and compare them with the work presented in Section 2.2 using the Messidor-2 dataset. Table 6 provides a summary of the performance metrics obtained by our system and previous works.

**Table 6:** Performance comparison with previous state-of-the-art works for referable DR detection on the Messidor-2 dataset. While our model is trained as a five-class grader, the metrics shown here are for the binary (referable vs. non-referable) task to ensure a fair comparison with prior studies, which are all binary classifiers.

Study	AUC (%)	Sensitivity (%)	Specificity (%)	Accuracy (%)
Pires et al. [35]	98.2			
Gargeya et al. [36]	94			
Abràmoff et al. [33]	93.5	96.5	59.4	
Abràmoff et al. [38]	98	96.8	87	
Gulshan et al. [37]	99	97.5	93.4	
Voets et al. [40]	80	73.7	79.7	
Lahmar et al. [39]				85.8
Papadopoulos et al. [43]	97.6	95.4	84.5	
Zhang et al. [42]	91.8	90.2	93	
This work	98	98	96	96.7

It is important to note that all these works perform a binary classification. In most cases, classifiers are used to detect referable DR, that is, DR cases that correspond to class 2 or higher according to the ICDR classification. However, in [36] the classification is performed between NoDR and all classes with any degree of DR. It is important to highlight that Table 6 provides only a limited view of the real advantages of our approach as it performs a compassion mostly with systems trained as two class classifiers without explanation capabilities or quality of the results indications.

All works except [39] use AUC as a classifier quality indicator. In [39] only the accuracy is used as a performance metric, and this is the reason we have included this parameter in Table 6. Sensitivity and specificity are also provided in all works except [35, 36, 39]. Analyzing Table 6 we can see that with regard to AUC our system outperforms all previous works except [35] and [37]. In the case of [35], this work does not provide any other metric other than AUC. In the case of [37], the reproduction study carried out in [40] produced considerably lower results. Regarding sensitivity and specificity, our system produces better results than previous work that used the Messidor-2 dataset. Regarding accuracy, our system also produces better results than [39] which is the only work that evaluates the classifier using this parameter.

The implications of this study extend beyond achieving state-of-the-art metrics and represent a significant shift in the design philosophy for AI-based diagnostic aids in ophthalmology. The key research implications are:

1. **A Paradigm Shift from Screening to Diagnostic Grading:** The vast majority of previous works focus on binary, referable vs. non-referable classification, positioning their tools primarily for screening [33, 35, 37, 38]. Our work’s primary implication is the successful development of a five-class ICDR classifier that provides granular diagnostic information. This is a crucial difference, as it moves the utility of the AI tool from a simple referral gateway to a genuine diagnostic assistant that can help ophthalmologists determine specific treatment and management plans based on a precise severity grade.
2. **Enhanced Clinical Trust through Multi-Faceted Explainability:** While some prior systems offer basic interpretability, they typically provide a single, static explanation. Our approach is fundamentally different by providing a set of configurable, superimposed heatmaps derived from the different ensemble networks. The implication is a more transparent and trustworthy system. As our results show, ophthalmologists may prefer different explanations for different cases, and providing this flexibility allows the tool to better integrate into the clinical workflow and build confidence with the practitioner.
3. **Introduction of Quantitative Confidence for Nuanced Decision-Making:** Standard classifiers output a prediction, but our work introduces a novel “quality factor” to quantify the model’s confidence between the top two alternatives. The significance of this feature is its direct impact on clinical utility. It allows a clinician to immediately gauge the certainty of a result and pay closer attention to those where the model indicates low confidence or a close second option, directly improving the tool’s practical usability.

Collectively, these implications—a focus on diagnostic grading, interactive explainability, and quantitative confidence—outline a more clinically integrated and powerful framework for AI in diabetic retinopathy care.

The study has several limitations that should be considered. Firstly, there may be dataset bias due to the reliance on the Messidor-2 dataset and a subset of non-mydriatic images from the Kaggle EyePACS dataset, which could limit the model’s generalizability to images captured under different conditions. Additionally, the dataset size may not be sufficient to capture all possible variations of diabetic retinopathy, potentially affecting the model’s robustness. There is also an inherent imbalance in class distribution, with fewer samples for more severe stages, which could skew predictions despite the use of mitigation techniques. Another limitation is the generalization of model performance from clinical-grade images to real-world settings, where images might be of lower quality. While heatmaps are used to improve model interpretability, the explanations might not always align with clinical reasoning. However, this explainability issue is partly mitigated by providing several alternative explanations for clinicians to choose from, enhancing the support for decision-making. Lastly, the study’s use of pretrained models means that any biases or limitations in the base architectures could be inherited, affecting the overall performance and optimization for diabetic retinopathy detection. The analysis of the statistical significance of ophthalmologists’ preferences for different heatmap alternatives requires a pivotal clinical essay where a small group of ophthalmologists to evaluate the system in their daily work.

## Conclusions and future work

Our work represents a significant advancement by supporting the full five-class International Clinical Diabetic Retinopathy (ICDR) grading system, in contrast to many previous state-of-the-art models that primarily focus on binary classification for screening purposes. While our system demonstrates comparable or superior performance on standard metrics when benchmarked against these binary classifiers (as shown in Table 6), its primary contribution lies in providing much richer, diagnostically relevant information to ophthalmologists—moving beyond a simple “referable” or “non-referable” screening result to specific diagnostic grades.

The clinical utility of our model is enhanced by several key innovations designed for diagnostic confidence and reliability:

- **Clinically Aware Error Profiles:** The model demonstrates robust and predictable behavior, as classification errors are consistently confined to adjacent severity levels (as seen in the confusion matrix in Fig. 6), which is a crucial feature for minimizing diagnostic risk.
- **Quantitative Confidence Score:** We introduce a novel “quality indicator” that quantifies the system’s confidence in its prediction and explicitly identifies the second most probable class. This provides clinicians with critical context to better assess borderline or ambiguous cases.
- **Multi-Faceted Explainability:** Going beyond a single explanation, our classifier provides the ophthalmologist with a set of configurable, superimposed heatmaps. These visualizations correspond to the analyses of different networks within the ensemble, offering multiple perspectives on the anatomical regions influencing the diagnosis.

Together, these contributions create a more comprehensive, transparent, and clinically integrated diagnostic tool that is designed not just for screening, but for detailed grading and enhanced diagnostic support.

As a future work, we would like to train our system, as demonstrated in other studies, with either the full Kaggle EyePACS dataset or with the part of that dataset acquired with non-mydriatic fundus camera. The first option includes many images with characteristics that are very different from the Messidor-2 dataset, while the second option provides a smaller subset of Messidor-2 compatible images. We would compare the results on the full Messidor-2 dataset with those obtained in this work that uses part of the Messidor-2 complemented with a small subset of non-mydriatic fundus camera images from Kaggle EyePACS for training and the rest of the Messidor-2 dataset for validation.

Another area that requires future study is a DL system to automatically produce the best explanation among the possible alternatives. This will require creating a dataset where a significant number of explanations are rated by retina specialists.
